# Cost-effectiveness analysis of a hand hygiene monitoring system in a tertiary pediatric hospital in Mexico

**DOI:** 10.3389/fpubh.2023.1117680

**Published:** 2023-03-09

**Authors:** Guillermo Salinas-Escudero, Daniela De la Rosa-Zamboni, María Fernanda Carrillo-Vega, Ana Estela Gamiño-Arroyo, Filiberto Toledano-Toledano, Fernando Ortega-Riosvelasco, Víctor Granados-García, Mónica Villa-Guillén, Juan Garduño-Espinosa

**Affiliations:** ^1^Center for Economic and Social Studies in Health, Hospital Infantil de México Federico Gómez, Mexico City, Mexico; ^2^Subdirector Comprehensive Patient Attention, Hospital Infantil de México Federico Gómez, Mexico City, Mexico; ^3^Research Department, Instituto Nacional de Geriatría, Mexico City, Mexico; ^4^Hospital Epidemiology Department, Hospital Infantil de México Federico Gómez, Mexico City, Mexico; ^5^Unidad de Investigación en Medicina Basada en Evidencias, Hospital Infantil de México Federico Gómez, National Institute of Health, Mexico City, Mexico; ^6^Unidad de Investigación Sociomédica, Instituto Nacional de Rehabilitación Luis Guillermo Ibarra Ibarra, Mexico City, Mexico; ^7^Dirección de Investigación y Diseminación del Conocimiento, Instituto Nacional de Ciencias e Innovación para la Formación de Comunidad Científica, INDEHUS, Mexico City, Mexico; ^8^Head of Hospital Epidemiology Department, Hospital Infantil de México Federico Gómez, Mexico City, Mexico; ^9^Epidemiological and Health Services Research Unit Aging Area, Centro Médico Nacional, Mexico City, Mexico; ^10^Medical Director, Hospital Infantil de México Federico Gómez, Mexico City, Mexico; ^11^Research Director, Hospital Infantil de México Federico Gómez, Mexico City, Mexico

**Keywords:** hand hygiene, infection control and prevention, cost-effectiveness, infection rate per, 1,000 patient days, automated hand hygiene monitoring system (AHHMS)

## Abstract

**Background:**

An automated hand-hygiene monitoring system (AHHMS) was implemented in October 2019 at the *Hospital Infantil de México Federico Gómez* (HIMFG), a tertiary pediatric referral hospital, in four of the hospital wards with the highest rates of Healthcare Associated Infections (HAIs). The clinical and economic impact of this system had not yet been assessed prior to this study. This study aimed to evaluate if the AHHMS is a cost-effective alternative in reducing HAIs in the HIMFG.

**Methodology:**

A full cost-effectiveness economic assessment was carried out for the hospital. The alternatives assessed were AHHMS implementation *vis-a-vis* AHHMS non-implementation (historical tendency). The outcomes of interest were infection rate per 1,000 patient-days and cost savings as a result of prevented infections. Infection rate data per 1,000 patient-days (PD) were obtained from the hospital's Department of Epidemiology with respect to the AHHMS. As regards historical tendency, an infection-rate model was designed for the most recent 6-year period. Infection costs were obtained from a review of available literature on the subject, and the cost of the implemented AHHMS was provided by the hospital. The assessment period was 6 months. The incremental cost-effectiveness ratio was estimated. Costs are reported in US Dollars (2021). Univariate sensitivity and threshold analysis for different parameters was conducted.

**Results:**

The total estimated cost of the AHHMS alternative represented potential savings of $308,927–$546,795 US Dollars compared to non-implementation of the system (US$464,102 v. US$773,029–$1,010,898) for the period. AHHMS effectiveness was reflected in a diminished number of infections, 46–79 (−43.4–56.7%) compared to non-implementation (60 v. 106-139 infections).

**Conclusion:**

The AHHMS was found to be a cost-saving alternative for the HIMFG given its cost-effectiveness and lower cost *vis-a-vis* the alternate option. Accordingly, the recommendation was made of extending its use to other areas in the hospital.

## Introduction

Level 1A of Evidence for Clinical Practice Guidelines (1, 2) establishes that hand hygiene (HH) prevents Healthcare Associated Infections (HAIs), these being among the costliest health care risks as regards lives and financial resources worldwide (3). Despite all evidence published in the last two centuries on this subject, and notwithstanding that it has become a worldwide standard for patient safety (4), optimal full adherence to HH protocols at hospitals is still a complex task (5, 6). Performing HH as recommended by WHO guidelines requires not only behavioral changes from health care workers (HCWs), training and constant assessment and feedback, but also an institutional environment that promotes safety by the placement of reminder posters at the workplace. Various studies have reported that hand cleansing is a cost-effective strategy (7–10).

Systems to foster, monitor and improve HH compliance have been developed; among these electronically assisted/enhanced or video monitoring direct surveillance systems, electronic dispenser counters, and automated HH monitoring networks (11). Automated HH monitoring (AHHM) systems offer the advantage of providing constant and individualized feedback on protocol compliance. Generally, an AHHMS includes personalized ID tags for each health care worker; this device establishes wireless contact with alcohol-rub dispensers, after which the information is relayed to a central system that measures HH performance in real time. The success of AHHM systems in improving HH performance has been reported in a number of countries (3), but very little has been said in regard to their impact on HAI rates, as focus has been directed towards assessing compliance, an intermediate stage in the ultimate desired outcome of HAI reduction, which is the main purpose of all HH programs. Consequently, there is no clear evidence as yet showing that the implementation of an AHHMS is a cost-effective alternative for hospitals and health care facilities.

Although Guest FJ. (12) published an assessment based on an economic model, the study takes into consideration theoretical HAI reductions, and does not take into account efficacy and effectiveness data. A study by S. McCalla reports a significant reduction in HAI infection rates, in catheter-associated urinary tract infections: IRR of 0.55, 95% CI, 0.35–0.87, and in central line-associated bloodstream infections: IRR of 0.45; 95% CI, 0.23–0.89 (13). This study aimed at assessing if the “AIDY” AHHMS implemented at the Hospital Infantil de México Federico Gómez (HIMFG) in October 2019 was a cost-effective technological strategy which contributed to the reduction in nosocomial HAIs.

## Methodology

A full cost-effectiveness economic assessment on the implementation of the AHHMS was carried out for the hospital. Both the clinical and economic impacts of this technology were evaluated. The compared inputs in the analysis were the AHHMS (intervention) against manual or traditional monitoring (control).

The HIMFG is a public pediatric tertiary referral hospital located in Mexico City. The hospital has well-established HH-compliance protocols which have rendered good outcomes (5). The AHHMS was implemented for all beds in four hospital wards which were chosen on the basis of internet availability, and for all resident physicians and nurses.

The AHHMS was implemented in wards 1 and 2 in October 2019, and in January and February 2020 in wards 3 and 4. Monthly records from all of the wards were obtained until March 2020 when data gathering was interrupted as hospital facilities were subject to COVID-19 reconversion due to the SARS-CoV-2 pandemic outbreak, which prevented gathering of HH unbiased measurements of adherence levels and hospital HAI rates due to the special COVID 19 HH protocols.

### Effectiveness

Effectiveness in this analysis was measured as the infection rate per 1,000 Patient Days (PD). Effectiveness of the intervention group was the infection rate reported by the Department of Epidemiology of the hospital for the 2019–2020 period analyzed. This rate was estimated on the basis of the information obtained from wards 1 and 2 for up to 6 monthly monitored opportunities. As regards wards 3 and 4, the length of the observation period was shorter.

Effectiveness of the control group (non-implementation option) was the infection rate per 1,000 PD as estimated by regression models. A polynomial regression model was used for each ward. Expected infection rates were calculated on the basis of the tendency of the previous six-year period from 2013 to 2019 (during the same months taken into account for the intervention).

The estimated infection rate allowed us to calculate the potential number of infections for the non-implementation alternative for each ward, and thus allowed us to make the assessment in terms of the resulting infection rate per 1,000 hospitalized patients and in terms of prevented infections.

Due to the departure from normality of the infection rate data of all wards (Shapiro-Wilk test), polynomial regressions were applied to the 2013–2019 data to achieve good expected predictive values for the 2019–2020 infection rates with a minimum regression coefficient (R2) of 84%. The infection-treatment cost used in the model was US$7,288.37 (14) for a tertiary pediatric hospital in Mexico, adjusted for inflation as of December 2021 (15), expressed in US Dollars [at an exchange rate of $20.46 Pesos Mex. Cy. per US$1.00 (16)]. The cost of the “AIDY” AHHMS reported by the HIMFG is US$1.64 per day per bed. Costs of hand-cleansing agents (such as alcohol rub, soap, water etc.) were not included in the analysis as these are available throughout the hospital as part of its “Let's hit 100” HH program (5), do not depend on the automated monitoring program and should therefore not affect the relationship between the relative costs of the two alternatives.

Approval from the Research and Ethics Committee of the HIMFG for the study was obtained and recorded under registration numbers HIM/2015/048 and HIM/2017/134. Data used in the analysis did not include sensitive information of the patients treated in the hospital during the study period and were restricted to the records provided by the hospital Epidemiology Department.

### Cost-effectiveness model

A deterministic mathematical model was constructed for the baseline case which was represented by a decision tree to compare the two management alternatives: AHHMS intervention group v. the non-intervention group (manual/regular surveillance).

**Figure 1:**
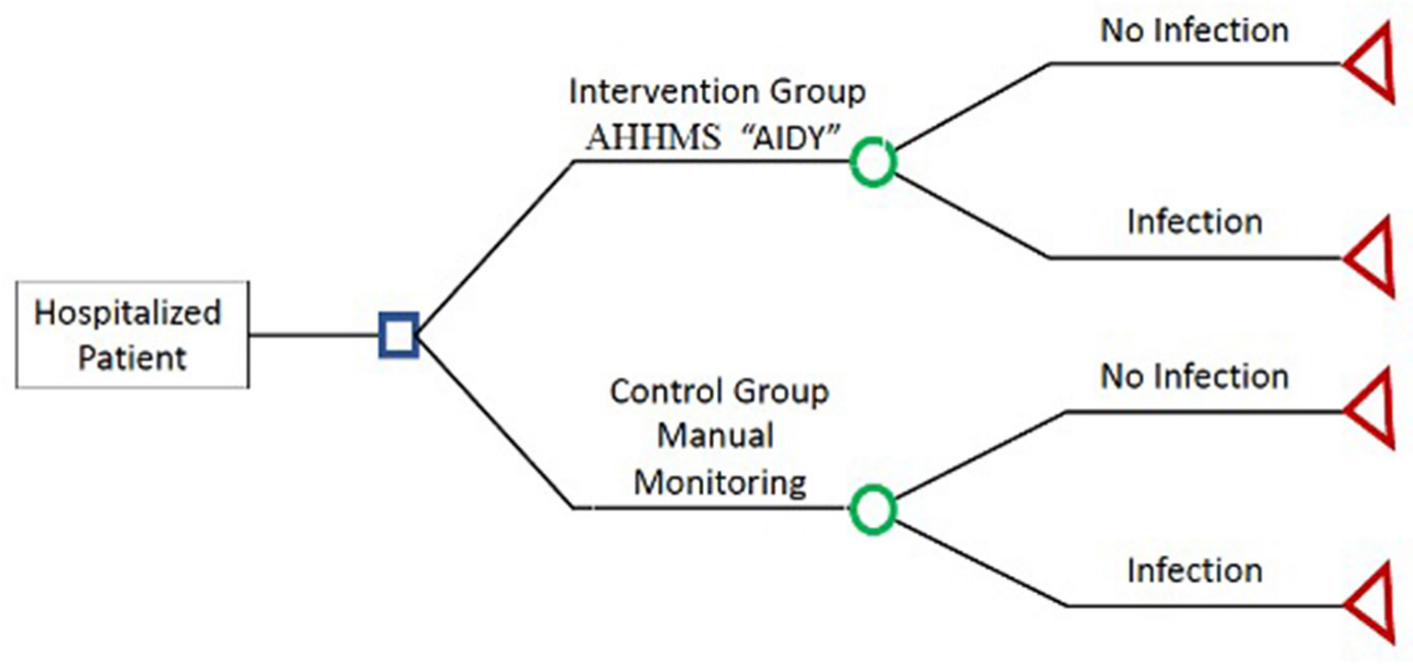
Cost effectiveness decision tree.

The model was built for a cohort of patients admitted at the HIMFG who were provided care under two scenarios: one in which an AHHMS was used, and the other where healthcare was provided under the traditional or regular HH surveillance system. Infection rates were determined on the basis of actual infections occurring while the AHHMS was in use, and for the traditional scenario, the rates were those estimated on the basis of infection rates per 1,000 PD for each of the alternatives (Figure 1).

The time horizon for the economic evaluation was set at 6 months, and therefore a discount rate was not applied to health outcomes nor was it applied to costs. The expected model outcomes were global costs of each one of the alternatives. For the intervention group the cost of the automated program plus the cost of the reported infections were considered. As regards the control group, the sole cost taken into consideration was the healthcare costs of the infections occurring during the study period. The incremental cost-effectiveness ratio (ICER) was calculated by dividing incremental cost (Costs 1-Costs 2) by the effectiveness (Effectiveness1—Effectiveness2) of each of the compared alternatives, where alternative 1 is the AHHMS v. alternative 2 the traditional surveillance system.

### Sensitivity analysis

Threshold univariate sensitivity analysis was conducted for two scenarios contemplated, to ascertain the robustness of the main parameters of the model base case where greater uncertainty was assumed.

The first scenario involved the assessment of the cost per infection, as per the effectiveness reported by the AHHMS and the number of prevented infections at which the implementation of this program ceased to be economically advantageous for the HIMFG.

In this scenario, cost for the period for each ward with the AHHMS intervention was divided by the number of prevented infections and then compared to the number of infections occurring under the non-intervention alternative.

The second scenario also involved sensitivity analysis to assess which was the lowest number of infections in the base case (the cost per bed of the AHHMS remaining constant) at which the AHHM program ceased to be economically advantageous for the HIMFG. In this second scenario, cost for the period for each ward with the AHHMS intervention was divided by the cost of infection considered in the base case.

Computations were done on Microsoft Excel and cost-effectiveness analysis was conducted with TreeAge Pro v. 2009.

## Results

Table 1 shows HAI rates per 1,000 PD for the wards under study, the 2019–2020 values are those recorded at the time the “AIDY” AHHMS was implemented. Central tendency measures (mean and SD) can be appreciated. Ward 2 presented the highest HAI rate, 7.3 infections/1000 PD (SD 3.3), followed by wards 3, 1, and 4.

**Table 1:** Characteristics of the HIMFG wards 2013–2020.

**Area**	**Healthcare service included**	**Number of beds**	**Intervention duration (months)**	**HAI rate per 1,000 PD 2013–2019**	**Variable**	**Period**						**AIDY AHHMS**	**Mean**	**Std. dev**.
						**13–14**	**14–15**	**15–16**	**16–17**	**17–18**	**18–19**	**19–20**		
Ward 1	Endocrinology	22	6	4.4	Infections	14	15	20	17	26	28	14		
	Infectology				Patients-days	4,566	4,484	4,583	4,298	4,268	4,766	3,801		
	Internal medicine				Infection rate	3.1	3.3	4.4	4.0	6.1	5.9	3.7	4.4	1.2
Ward 2	Sundry pediatric care	30	6	7.3	Infections	25	29	12	23	23	36	22		
	Neurology													
	Pulmonology							Patients-days	4,243	3,170	3,916	4,287	3,400	2,666	2,661		
					Rheumatology												
	Cardiology				Infection rate	5.9	9.1	3.1	5.4	6.8	13.5	8.3	7.3	3.3
	Gastroenterology-nutrition													
Ward 3	Surgery	56	2	6.0	Infections	10	15	9	13	18	11	12		
	Nephrology				Patients-days	1,895	1,980	2,216	2,184	2,290	2,189	1,940		
					Infection rate	5.3	7.6	4.1	6.0	7.9	5.0	6.2	6.0	1.4
Ward 4	Hematology	40	3	3.5	Infections	11	14	13	8	10	20	12		
	Oncology				Patients-days	2,966	3,639	3,939	3,999	3,798	3,799	3,629		
					Infection rate	3.7	3.8	3.3	2.0	2.6	5.3	3.3	3.5	1.0
Total					Infections	60	73	54	61	77	95	60		
					Patients-days	9,427	10,103	10,738	10,481	10,356	10,754	9,370		
					Infection rate	6.4	7.2	5.0	5.8	7.4	8.8	6.4	6.71	1.22

PD, Patient days.

Table 2 shows the polynomial equations used to obtain infection-rate projected values during the 2019–2020 period for the four wards, as well as regression coefficient R2 and the projected value for the model for the same period.

**Table 2:** Polynominal equations.

**Area**	**X0**	**X1**	**X2**	**X3**	**X4**	**X5**	**R2**	**Projected value**
Ward 1	2.262	0.625					0.843	6.6
Ward 2	−15.993	38.066	19.544	3.771	−0.239		0.891	11.8
Ward 3	−47.896	105.540	70.660	20.998	2.846	0.144	1.0	10.1
Ward 4	10.831	−4.520	0.592				0.863	8.2
Total	6.5977	0.4153	0.4296	0.0714			0.76	12.9

When comparing the results of projected infection rates v. the values obtained for the AIDY AHHMS for the 2019–2020 period for the different wards, as well as the number of prevented infections with the AIDY AHHMS intervention (Table 3), a general reduction in the number of infections is observed. This difference was statistically significant (*P* < 0.01) with the *Z*-test used to establish rate differences for all wards.

**Table 3:** Infection rates.

**Area**	**“AIDY” AHHMS 19_20**	**Projected**			**Rate diff AIDY vs. projected**	***P***	**Prevented infections AIDY vs. projected**
		**19_20**	**Min**	**Max**			
Ward 1	3.7	6.6	3.8	9.4	2.9	*	11.2
Ward 2	8.3	11.8	4.5	19.1	3.5	*	9.4
Ward 3	6.2	10.1	6.0	14.2	3.9	*	7.7
Ward 4	3.3	8.2	3.8	12.6	4.9	*	17.8
Global	6.4	12.9	7.8	18.1	6.54	*	78.7

^*^Z-test (P < 0.01).

The cost of the AHHM program ranged from $5,409 to $8,939 during the study period for each of the different wards, depending on the number of beds and the time the beds were included, representing a total cost of $26,800 for the four wards as shown in Table 4.

**Table 4:** Costs of the alternatives.

**Area**	**Start date**	**End date**	**Total days**	**Beds**	**AIDY 19_20 infections**	**Control 19_20 infections**	**Infection difference**	**Bed daily cost**	**AIDY program cost**	**Infection cost**	**Total AIDY cost**	**Total control group**	**Program Net savings**
Ward 1	01/10/19	31/03/20	182	22	14	25	11	$1.64	$6,555.91	$7,288.37	$108,593.16	$183,671.63	$75,078.47
Ward 2	01/10/19	31/03/20	182	30	22	31	9	$1.64	$8,939.88	$7,288.37	$169,284.12	$229,039.68	$59,755.56
Ward 3	01/02/20	31/03/20	59	56	12	20	8	$1.64	$5,409.78	$7,288.37	$92,870.27	$143,328.74	$50,458.47
Ward 4	01/01/20	31/03/20	90	40	12	30	18	$1.64	$5,894.43	$7,288.37	$93,354.92	$216,989.14	$123,634.22
Total							46^*^/78.7^**^		$26,800.00		$464,102	$773,029^*^/ $1,010,898^**^	$308,927^*^/ $546,795^**^

^*^Obtained directly by the sum of the four rooms.

^**^Number of infections prevented obtained using the polytomous regression model.

Total cost for the AHHM alternative, which included both the cost of the program and infection treatment costs were in the range of $92,870 to $169,284, representing a global cost for the hospital of $464,102. In regard to the non-intervention alternative, costs ranged from $143,328 to $229,039, depending on the ward, representing a global expenditure for the hospital of $773,029. Thus, implementation of the AHHM program resulted in net savings in the range of $50,458 to $123,634; and an amount of $308,629 in savings during the study period.

### Cost-effectiveness outcomes (base case)

In assessing total costs for each alternative, we found that such costs for implementing the AHHM program in the four wards were lower than those of the non-implementation alternative for the study period (Table 5), resulting in savings in the $50,458 to $123,634 range. With respect to effectiveness, we observed that the number of infections in all wards was lower in the AHHM intervention group when compared to the non-intervention group, keeping in mind that best outcomes are those with the lowest number of infections. Finally, ICER in all wards of the non-intervention group was markedly surpassed by that of the AHHM, as the latter's outcomes were lower numbers of infections at lesser cost for the HIMFG making it a cost-saving alternative.

**Table 5:** Cost effectiveness results.

**Strategy**	**Cost**	**Effectiveness**	**Incremental cost**	**Incremental effectiveness**	**RCEI**
**Ward 1**					
AHHMS AIDY	$108,593	14			Dominant
No intervention	$183,671	25	$75,078	−11	Dominated
**Ward 2**					
AHHMS AIDY	$169,284	22			Dominant
No intervention	$229,039	31	$59,755	−9	Dominated
**Ward 3**					
AHHMS AIDY	$92,870	12			Dominant
No intervention	$143,328	20	$50,458	−8	Dominated
**Ward 4**					
AHHMS AIDY	$93,354	12			Dominant
No intervention	$216,989	30	$123,634	−18	Dominated
**Total** ^*^					
AHHMS AIDY	$464,102	60			Dominant
No intervention	$773,029	106	$308,927	−46	Dominated
**Total** ^**^					
AHHMS AIDY	$464,102	60			Dominant
No intervention	$773,029	139	$546,795	−79	Dominated

^*^Obtained directly by the sum of the four rooms.

^**^Number of infections prevented obtained using the polytomous regression model.

If the global sum of infections prevented for the four HIMFG wards is used as the total scenario, the program had a cost of US$ 464,102 vs. the non-implementation of US$773,029. The implementation reduced 46 infections (43.4%) (60 vs. 106), resulting in the hospital's net saving of US$308,927. If the estimate of the infection rate is obtained through global polytomous regression, the program had a cost of US$464,102, while a non-implementation cost of US$1,010,898. The program reduced 79 infections (56.7%) (60 vs. 139), resulting in net savings for the hospital of US$546,795, so both results allow considering the program's implementation as a cost-saving alternative.

### Sensitivity analysis results

Two scenarios were established to assess robustness of the main parameters of the base model.

#### Scenario 1

The minimum cost per infection for the different wards was $331 (ward 4), $585 (ward 1), $705 (ward 3) and $948 (ward 2), with an average minimum cost per infection for the hospital of $582, taking into account a total of 46 prevented infections and the global implementation cost of the AHHM alternative.

#### Scenario 2

The minimum number of prevented infections required to offset the cost of the AHHMS vis-à-vis non-implementation of the system in the different wards was: 0.82 (ward 2), 1.11 (ward 1), 1.24 (ward 4) and 1.35 (ward 3), the overall minimum of prevented infections for the HIMFG for the period being 3.68.

## Discussion

Our results showed that the AHHMS was a cost-effective and cost-savings alternative in reducing infection rates per 1,000 PD, and in reducing HAIs, representing substantial savings for the HIMFG. This is the first study showing the cost-effectiveness of an AHHMS in comparison with manual monitoring which we conducted by shadowing techniques, since the results reported by Guest et al. were based on effectiveness data of other studies (12). Although implementation of an AHHMS may entail a significant investment for healthcare providers as reported in other studies (17); our results show that the return obtained as a consequence of the reduction in infections is greater and may even result in savings.

Daily costs of AHHM per bed, reported by us for the HIMFG closely resemble those reported internationally ($450–$650 per annum) (18), thus we conclude that there is no bias in our results. However, healthcare facilities may strive to obtain a reduction in cost from the supplier to obtain better outcomes, provided the quality of both the service and equipment monitoring is not sacrificed.

Our results on the effectiveness of an AHHMS in reducing infections differ from those reported in previous works on the subject (19), since the latter all mention that various additional measures need to be implemented together with the system. However, as the HIMFG does have a well-established HH program, our results serve to provide evidence of the potential benefits to be obtained from implementing an AHHMS in enhancing permanent and interactive compliance, while providing proof of its impact on infection reduction. As reported by McCalla et al., we did observe a positive impact (decline) in the number of infections (13).

Our study was subject to certain time limitations, as we were unable to monitor the intervention due to the COVID-19 pandemic, which modified healthcare protocols and forced us to halt monitoring as potential biases would have affected new infection rates estimates for the different hospital wards. Although there is evidence that the COVID-19 outbreak modified HH adherence (20), there is no clear evidence that it had an effect on the number of infections, though the likelihood of such impact is to be expected.

Another limitation was the lack of cost estimates of pediatric infections for the HIMFG in particular. Such estimates would have been an ideal complement for our study. Nevertheless, the costs taken into account for our model were conservative if compared to other costs reported for the adult population in Mexico (21) or internationally (22).

With respect to the sensitivity analysis conducted, the parameters employed in estimating costs and effectiveness were consistent, given that the values used were conservative and allowed for the assumption of extreme values of much lower effectiveness, or significantly smaller infections costs, and the results in both instances would have nevertheless provided favorable outcomes in the implementation of the AHHMS at the HIMFG.

We expect to analyze infection rates when the COVID-19 pandemic in over at the HIMFG, once healthcare protocols are again fully in place, to assess long-term outcomes when AHHM is implemented in other areas of the hospital.

## Conclusion

The AHHMS proved to be a cost-saving alternative for the HIMFG given its greater effectiveness and lower costs when compared to the present one. Accordingly, the recommendation was made to extend the AHHMS to the remaining hospital wards with similar internet access and comparable infection rates.

## Data availability statement

The original contributions presented in the study are included in the article/Supplementary material, further inquiries can be directed to the corresponding author.

## Ethics statement

The studies involving human participants were reviewed and approved by Research and Ethics Committee of the HIMFG for the study was obtained and recorded under Registration Numbers HIM/2015/048 and HIM/2017/134. Written informed consent to participate in this study was provided by the participants' legal guardian/next of kin.

## Author contributions

Conceptualization and formal analysis: GS-E and DR-Z. Data curation and supervision: GS-E, DR-Z, AG-A, and FO-R. Investigation: GS-E, MC-V, and MV-G. Methodology: GS-E, DR-Z, AG-A, FO-R, JG-E, and VG-G. Writing—original draft: GS-E, DR-Z, MC-V, AG-A, and FO-R. Writing—review and editing: GS-E, DR-Z, MC-V, AG-A, FT-T, FO-R, VG-G, MV-G, and JG-E. All authors contributed to the article and approved the submitted version.
