# Supplementary material for: Cost-effectiveness analysis of a hand hygiene monitoring system in a tertiary pediatric hospital in Mexico
Source: Front Public Health. 2023 Mar 9;11:1117680. doi: 10.3389/fpubh.2023.1117680 (PMC10034395; doi:10.3389/fpubh.2023.1117680)
Supplement: Supplementary file 1 [file Table_1.DOCX]

Supplementary Material

Cost-effectiveness analysis of a hand hygiene monitoring system in a tertiary pediatric hospital in Mexico

**Guillermo Salinas-Escudero, Daniela De la Rosa-Zamboni*, María Fernanda Carrillo-Vega, Ana Estela Gamiño-Arroyo, Filiberto Toledano-Toledano, Fernando Ortega-Riosvelasco, Víctor Granados-García, Mónica Villa-Guillén, Juan Garduño-Espinosa**

*** Correspondence:** Mtra. Daniela De la Rosa-Zamboni: rzdaniela@hotmail.com

# Supplementary Data

AIDY is an electronic device designed to foster HH adherence, created in 2015 in which several technological trends converged. Every healthcare worker must carry a wearable identification tag worn or attached to the upper torso. To detect HH opportunities, AIDY incorporates a real-time locating system (RTLS), capable of recording every instance in which a healthcare worker wearing the AIDY tag comes in contact with a patient or his environment (Beacon). The use of hand-sanitizer dispensers is recorded by adding a sensor (smart-dispenser) via the internet of things (IoT). All the data generated by the system are uploaded to the cloud and analyzed in real time. Reports are then shown on a screen or downloaded to a web application, which can be accessed from any mobile device with internet access. AIDY devices use low-energy Bluetooth to connect to the Internet through Wi-Fi 802.11, all systems communications take place at a frequency of 2.4 GHz, providing full security for hospital use. This is information as reported by the service supplier.(Aidy Technology, no date)

**
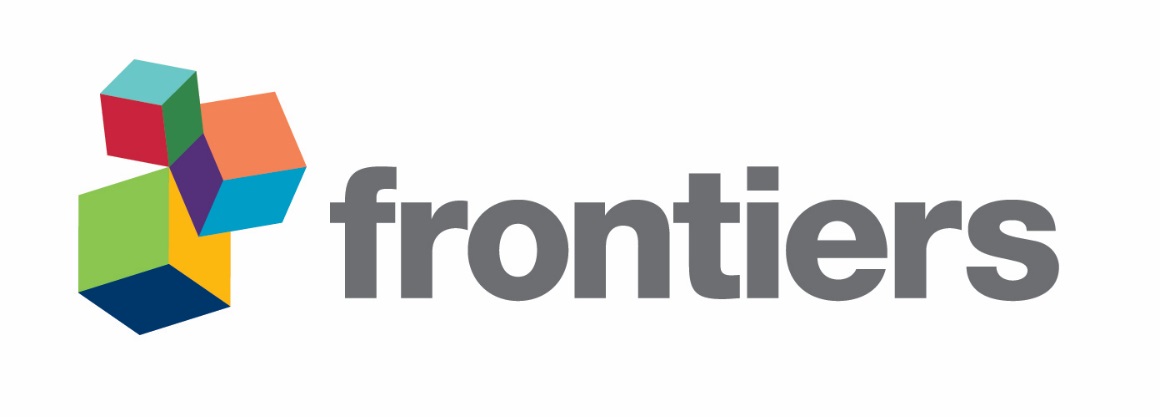
**
